# Exploring magnetic resonance imaging validation of length-based scaling of musculoskeletal models using OpenSim and AddBiomechanics for walking

**DOI:** 10.7717/peerj.21114

**Published:** 2026-04-22

**Authors:** Namra Rauf, Philipp Amon, Christoph Stuprich, Frederik B. Laun, Anne D. Koelewijn

**Affiliations:** 1 Chair of Autonomous Systems and Mechatronics, Department of Electrical Engineering, Friedrich-Alexander Universität Erlangen-Nürnberg, Erlangen, Germany; 2 Institute of Radiology, Uniklinikum Erlangen, Friedrich-Alexander Universität Erlangen-Nürnberg, Erlangen, Germany

**Keywords:** MRI, OMC, RMSE, OpenSim scaling, Personalized MSK models, AddBiomechanics, Joint angles, Participant-specific MSK models, OpenSim scaling

## Abstract

Scaling is an important step for achieving accurate participant-specific models when studying human motion. Scaling a generic musculoskeletal model using OpenSim is time consuming, depends on the user expertise, and requires a static pose. Recently, AddBiomechanics introduced automatic scaling of the participant-specific models independent of user expertise and static pose. However, its validation is limited to synthetic data. In this exploratory study, we compared models scaled *via* AddBiomechanics and OpenSim against models scaled based on magnetic resonance images (MRI). We performed an optical motion capture experiment in which we recorded walking at 0.8 m/s, 1.2 m/s, and 1.6 m/s, followed by an MRI scan of the lower extremities for 10 participants (M/F). We scaled the model using OpenSim, AddBiomechanics, and using the MRI data. In OpenSim, it was necessary for some participants to lock certain joint coordinates before scaling to avoid unrealistic postures. To evaluate the different models, we performed inverse kinematics for the three different walking trials of each participant and analyzed the joint angle trajectories and overall average root mean square error (RMSE) between the measured markers and virtual markers of the models scaled with OpenSim, AddBiomechanics and MRI. For those participants where we did not lock coordinates in the OpenSim model, the average marker RMSE was 1.657 cm for the OpenSim model, compared to 1.585 cm with AddBiomechanics and 1.471 cm for the MRI-based model. For the participants where we locked coordinates, the RMSE was 1.588 cm for the OpenSim model, compared to 1.725 cm with AddBiomechanics and 1.439 cm for the MRI-based model. The joint angles were similar, with the largest difference for the models with locked coordinates, where the maximum difference was 9.2° (ankle angle). Our exploratory study suggests that AddBiomechanics offers a practical alternative to OpenSim, showing comparable accuracy with no meaningful differences, while requiring less time and user effort.

## Introduction

Musculoskeletal (MSK) modeling is used to represent the dynamics of the human body. These MSK models can output many different variables, such as joint angles, joint moments and muscle forces. These variables are important for understanding, predicting and reconstructing human motion. To acquire human motion data, an optical motion capture (OMC) experiment is performed in a laboratory setting. During an OMC experiment, reflective markers and multiple infrared cameras are used to record the position of the anatomical landmarks. In addition, ground reaction forces during the movement are recorded through force plates. This is the standard procedure for recording human motion data and quantifying human biomechanics (Wade et al., 2022; Topley & Richards, 2020).

The acquired human motion data can be processed by performing inverse kinematics and inverse dynamics with an MSK model. In inverse kinematics, the joint angles are quantified such that the difference between measured markers and virtual markers placed on an MSK model are minimized, so that the model most closely replicates the motion of the participant. These calculated joint angles, along with ground reaction force data, can later be used to calculate net joint forces and moments. Personalization of an MSK model is necessary to ensure that the marker locations and segment lengths match those of the experimental participant. There are different software packages available for performing these processes, such as OpenSim and Anybody, where OpenSim is most commonly used since it is freely available.

MSK models used for studying human motion are most accurate when they are personalized *via* imaging techniques, *i.e.,* MRI or computed tomography (CT). MRI can be used as a reliable and accurate technique for calculating segment length in living participants (Doyle & Winsor, 2011). The MRI-based personalized MSK models reveals substantial differences to the generic MSK models *i.e.,* models typically based on the average measurements from cadavers. These personalized models can be used to investigate and validate other aspects of MSK models. The magnitude and location of the contact pressure at the knee were found to change due to the variation in joint kinematics and joint moments between the OpenSim generic model and MRI-based personalized model (Killen, Willems & Jonkers, 2024). Personalization led to increased anatomical consistency among three ankle joint models, each model personalized at a different level (Conconi et al., 2021). Another study with MRI-based personalized models reported significant differences to the generic models in the ankle dorsi/plantar flexion angle and net joint moments. These personalized models demonstrated better torque matching, physiologically plausible fiber lengths, higher fiber velocities, lower muscle forces, and lower simulated activations. However, the creation of these models is expensive and comes with a high computational cost (Akhundov et al., 2022).

In comparison, participant-specific models that are created by scaling a generic musculoskeletal model to an individual’s anthropometric parameters can be created more cheaply and rapidly (Correa et al., 2011; Saraswat, Andersen & MacWilliams, 2010). Participant-specific MSK models can be scaled through different approaches. The scaling starts with the computation of scale factors for different body segments, either *via* manual scaling or measurement based scaling. These scale factors are used to scale the model’s geometry, mass and inertial properties of segments(in combination with the input mass and preserve mass distribution), muscles and other model components dependent on the length. In the multistage process of scaling in OpenSim, the software rebuilds a virtual replica of the experimental participant, the focus being that the segment dimensions of the virtual replica match the experimental participant as accurately as possible. In the next step after scaling the model, the model’s markers are moved to match the experimental marker locations in the static pose by solving an inverse kinematic problem. It is followed by marker registration during which the locations of some markers on the model are adjusted manually to match the experimental data. However, this complete process of scaling is quite time consuming because of the mutual dependence of scaling, inverse kinematics and marker registration (Delp et al., 2007). This process needs considerable refinement of the visual iterative “guess-and-check” process, which restricts the scalability, decreases the repeatability, and increases the time required (Bakke & Besier, 2020; Dunne et al., 2021; Sturdy, Silverman & Pickle, 2022).

To speed up scaling, different approaches have been developed to automate the process. Charlton et al. (2004) and Reinbolt et al. (2005) took the lead in introducing optimization to automate the scaling and registration process. Their proposed methods improved the repeatability, but they come with a high computational cost. These methods uses an inner optimizer to generate an iterative guess regarding the body segment scaling and marker offsets, and then uses a computationally costly inner optimization problem to evaluate the quality of the guess. This leads to high time related computational cost. More recently, the automatic scaling toolkit (AST) has been proposed to reduce the time required for scaling in OpenSim (Di Pietro et al., 2024). It performs scaling and inverse kinematics on a static trial to iteratively adjust the positions of the virtual markers by implementing corrective actions until desired accuracy in terms of markers RMSE is reached. AST tries to automate the operations that are otherwise done manually by a user to obtain a properly scaled model (Di Pietro et al., 2024). AddBiomechanics has also been introduced recently for automatic scaling (Werling et al., 2023). This open-source web-based software combines linear methods and bi-level optimization to swiftly automate scaling, marker registration and inverse kinematics. In first stage, linear methods are employed followed by non-convex bi-level optimization to scale the body segments of an MSK model, register the locations of OMC markers placed on an experimental participant to that on an MSK model, and calculate the kinematics of the body segment considering the trajectories of experimental markers during a motion. In the second stage, a linear method is applied followed by another non-convex optimization to calculate body segment masses and refine kinematics to reduce residual forces by considering the corresponding ground reaction forces. The proposed scaling algorithm of AddBiomechanics has been evaluated on the previously published walking and multiple-activity dataset of Hamner & Delp (2013), along with synthetic data (Werling et al., 2023). However, the performance of the models scaled by AddBiomechanics is not validated against an actual ground truth data, *i.e.,* MRI, CT *etc*.

In this study, we explore the validation of models scaled with marker data from both male and female participants in AddBiomechanics and OpenSim, compared to the personalized models using MRI. Through this validation method, our goal is to provide compelling evidence that can guide other researchers in selecting the most effective scaling approach between AddBiomechanics and OpenSim. We performed an OMC experiment in a lab followed by an MRI scan of the lower-limbs on the same day. We personalized the model in AddBiomechanics and OpenSim using only the OMC data. We also tested symmetric and asymmetric scaling in OpenSim for the different models. We additionally used the MRI and OMC data to create the MRI-based participant-specific model. Using the MRI-based personalized model as ground truth, we evaluated the personalization accuracy of AddBiomechanics and OpenSim and investigated considering the time and user effort involved, if the personalization through AddBiomechanics would lead to higher accuracy than personalization through OpenSim. A preprint version of this manuscript was previously published on SSRN (Rauf et al., 2025).

## Methods

### Data acquisition

Data were collected from ten healthy participants (five male and five female, ages 18-45 years, weight 73.07 ± 11.7 kg, and height 1.7 ± 0.1 m) with no previous neurological/muscular disease and no musculoskeletal injury. One participant was excluded from the study due to missing left ankle marker data. The experiment was approved by the ethical committee of the Friedrich-Alexander-Universität Erlangen-Nürnberg (Ref.-No. 20-471-B). The study was conducted in accordance with the local legislation and institutional requirements. The participants were informed about the experiment in advance and provided their written informed consent before participating in the study.

The gait experiment was performed in a laboratory with nine Oqus cameras (Qualisys AB, Gothenburg, Sweden) sampling at 200 Hz and an instrumented split-belt treadmill (Bertec Corporation, Columbus, OH, USA) sampling at 1,000 Hz. Reflective markers, adapted from Koelewijn, Heinrich & Van den Bogert (2019) with additional markers added, were used to record the participant’s motion. We first recorded a static T-pose (both arms outstretched and legs slightly apart) for 10 s. Then, we recorded three types of walking for 150 s, that is, slow walking at 0.8 m/s, normal walking at 1.2 m/s and fast walking at 1.6 m/s. During each trial, the participant was allowed to familiarize themselves with the speed of the treadmill for a short duration of time before the recording started.

MRI data were acquired using a 3 T scanner (Magnetom Prisma, Siemens Healthineers, Erlangen, Germany). Multiple 18-channel body coils were placed on the legs from ankle to hip to acquire the data. A 3D FLASH sequence weighted with T1-weighted was used (voxel size 0.7 × 0.7 × 1.0 mm^3^, number of slices –224 per segment, acquisition time per stack - 4:01 mins, TR - 5.48 ms, TE - 2.49 ms, bandwidth - 370 Hz/Pixel). The participants were asked to lie in a ‘feet first-supine’ position inside the scanner. A foam cushion was placed below the participant’s knees to keep the calf muscle belly from pressing against the scanner bed. To mimic a normal standing position, a vertical support was provided at both feet. The scanner scanned the participant from the belly button to the toes in a total of four or five stacks, depending on the size of participant.

### Data processing

We used Qualisys Track Manager (QTM 2021.2) to label the 3D trajectories of the markers and to perform interpolation of the missed trajectories. We exported the data in C3D format from Qualisys and converted those to Track Row Column files to be read by OpenSim and AddBiomechanics.

**Figure 1:**
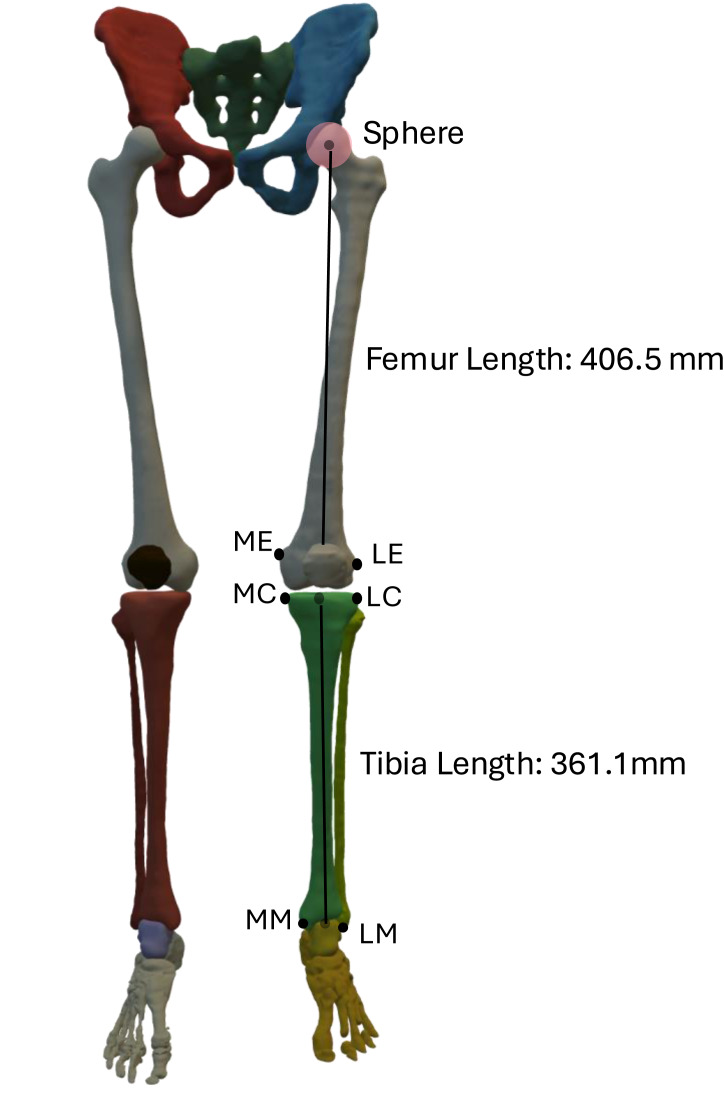
Coordinate planes defined through the anatomical landmarks, *i.e.,* medial condyle (MC) and Lateral condyle (LC), medial epicondyle (ME) and lateral epicondyle (LE), medial malleolus (MM) and lateral malleolus (LM).

The MRI scans of each participant were processed initially by converting the T1-weighted DICOM images to NIfTI images to ensure the anonymity of the participants. The images of the various segments of the lower limbs were acquired in stacks, and these stacks were manually combined and overlapping areas were removed. We performed semi-automatic segmentation of the bone tissue from the scans in 3D Slicer (Fedorov et al., 2012). Automatic segmentation was done using TotalSegmentator package (Wasserthal et al., 2023) followed by manual corrections using Gaussian filter. To avoid variations, same operator performed the corrections using a constant kernel size of three mm. We defined bony landmarks at the hip, knee and ankle joint according to the definition by Wu et al. (2002). To ensure consistency, the landmarks across all participants were defined by the same operator. The origin of the hip joint was defined as the center of a sphere that was fitted into the femoral head. The midpoint between medial and lateral epicondyles, and the tips of the medial and lateral malleoli coincide with the origin of femur and tibia at the knee respectively (Carbone et al., 2015). We defined the femur length as the Euclidean distance between knee and hip coordinate system, the tibia length as the Euclidean distance between the ankle and knee coordinate system, and the pelvis length as the Euclidean distance between the left and right hip joint origin, respectively (Fig. 1).

**Figure 2:**
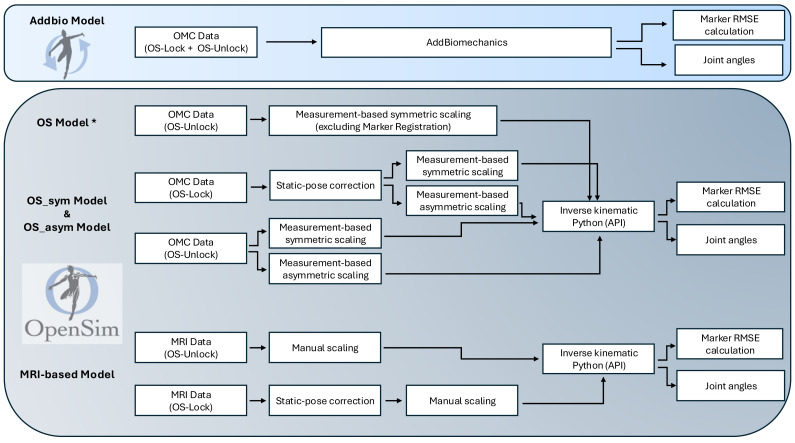
An overview of the different steps in the process of generating the five distinct personalized models. Addbio Model is created with AddBiomechanics and OS_sym Model (symmetric), OS_asym Model (asymmetric) and MRI-based Model are created with OpenSim. The Armless Rajagopal model was used as the standard generic model in all approaches. The gait data is divided into OS-Lock and OS-Unlock, based on the locking of certain joints before scaling in OpenSim.

We generated five distinct personalized models (Fig. 2). *AddBio model* was created with the web-based AddBiomechanics platform. *OS_sym model* and *OS_asym model* were created with OpenSim based on symmetric and asymmetric scaling. Since marker registration is time consuming, we also created the OS Model* for the symmetric model, by omitting marker registration and performing scaling *via* marker weights only. *MRI-based model* was generated based on the MRI data of the participants.

To create the *AddBio model* and perform inverse kinematics, we uploaded all trials of all participants to AddBiomechanics. This list contains the input parameters for the AddBiomechanics: height (m), mass (kg), sex, age, unimpaired, custom OpenSim upload model, and “do not fit physics”. The model described by Rajagopal et al. (2016) was used as our custom OpenSim model for AddBiomechanics. We chose the armless Rajagopal model because we had acquired the MRI for the lower extremities only. AddBiomechanics assumes symmetry between the right and left leg, similar to the principles of OpenSim symmetrical scaling.

In OpenSim, we performed measurement-based scaling *i.e.,* distances between virtual and model markers are compared to calculate respective scale factors for scaling a generic model. We used SACR, STRN, RShoulderTop, LShoulderTop, RAsis, LAsis, RKneeOut, LKneeOut, RAnkleOut, LAnkleOut, RHeelBack, LHeelBack, RToe and LToe for scaling (Appendix A). We performed symmetric scaling, where the left and right segments were used to generate one scaling factor, and asymmetric scaling, where a scaling factor was determined separately for the left and right side. Different marker weights were used for scaling and inverse kinematics depending on marker error value. The default convergence settings of inverse kinematics were used. To develop the OpenSim models, we initially categorized the OMC data into two groups, *OS-Lock* and *OS-Unlock*. *OS-Unlock* was composed of three participants while the *OS-Lock* was composed of six participants. The distinction was made because the OMC data of some participants after scaling led to larger marker errors, *i.e.,* RMS ≥ 3.7 cm, and unrealistic joint angles, *i.e.,* lumber flexion was ≥ 10 degrees when standing in the neutral pose. These values were identified as thresholding criteria for locking. A finely scaled model with lower marker error is necessary in the first step of scaling, otherwise these large marker errors propagates to the next steps *i.e.,* inverse kinematics or inverse dynamics. Therefore, for *OS-Lock*, scaling was performed while the static pose was corrected manually by adjusting torso’s height and locking certain joints, *i.e.,* pelvis tilt, pelvis list, lumbar extension, lumbar bending and right/left knee joints before the scaling process. The height of the torso was adjusted by manually assigning scale factors through the GUI, ensuring that the right and left shoulder markers were aligned correctly on the torso. For the *OS-Unlock*, no such adjustments were made. Then we created the *OS_sym model* (symmetric scaling) and *OS_asym model* (asymmetric scaling), by scaling the armless Rajagopal Model through an iterative guess-and-check process in OpenSim using the visual feedback from the static pose. The OS Model* was created only for the *OS-Unlock*.

To create the *MRI-based model*, we manually adjusted the reference model to each participant’s MRI data in OpenSim using the calculated body segment lengths of femur, tibia and pelvis. Moreover, as the feet were not automatically moved after changing the tibia length, the talus joint position was manually adjusted based on the change in tibia length to ensure that the feet were in the correct location. The upper body parameters remained unchanged with respect to the generic model. The model markers were fit to the model by using only the ‘Adjust Model Markers’ option in the scale tool.

### Analysis method

We evaluated the different scaling approaches using inverse kinematics on the walking trials. When a model is well-matched to a participant, the residual error between the measured markers and the virtual markers generated *via* inverse kinematics is expected to be smaller. Therefore, we used the root mean square error (RMSE) between the measured marker positions and the virtual marker positions found in the inverse kinematics for evaluating our results. Furthermore, we evaluated each scaled model by analyzing their joint angles, that is, ankle angle, knee angle and hip flexion angle for all the walking trials *i.e.,* slow walking, normal walking and fast walking. We performed inverse kinematics through OpenSim-Python API to calculate the virtual marker positions for the *OS_sym model*, *OS_asym model*, OS model *, and the *MRI-based model* for all the recorded trials. The inverse kinematics for the *Addbio model*, was executed automatically as part of the scaling process in AddBiomechanics.

We looked at the estimated lengths for the different body segments by OpenSim and AddBiomechanics, and compared them to the MRI-based lengths. To evaluate the scaling effect of locking, we separately evaluated the RMSE for the six participants, for which locking of joints was required (*OS-Lock*), and for the three participants, where it was not required (*OS-Unlock*). We also compared the RMSE between the experimental and virtual markers for all markers and separately for the markers on the legs, the markers on the torso, and the markers on the feet, as in the Appendix A. This separation was performed because the region containing the leg markers was specifically focused on during the MRI scan, and so we evaluated if the error was specifically lower in this region.

We also compared the RMSE of all experimental markers and the selective leg markers attached to the legs, torso markers attached to the torso and feet markers attached to the feet as in the Appendix A. The region containing the leg markers was specifically focused during the MRI scan.

## Results

**Figure 3:**
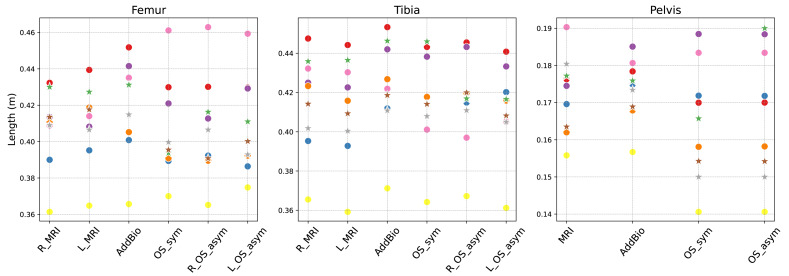
The lengths for various body segments estimated by AddBiomechanics and OpenSim models. (stars—*OS-Unlock* , dots—*OS-Lock*). OS_sym shows the symmetrical scaled model and OS_asym shows the asymmetrical scaled model. The R and L are the right and left body segment. The estimated lengths are compared to the actual lengths calculated from the MRI.

To evaluate the five scaled models, we first looked at the estimated scaled lengths of all these four models for the different body segments *i.e.,* right/left femur, right/left tibia and pelvis in Fig. 3. *OS-Unlock* in comparison to *OS-Lock*, shows that the average estimated femur length of the AddBiomechanics *i.e.,* 0.420 m, is closer to the average MRI-based lengths *i.e.,* 0.418 m (right) and 0.417 m (left), than the OpenSim lengths *i.e.,* asymmetric: 0.404 m (right), 0.401 m (left), symmetric: 0.396 m. The estimated average length of tibia by both OpenSim models *i.e.*,*OS_sym model* and *OS_asym model*: 0.417 m, asymmetric: 0.415 m (Right) 0.412 m (Left) was closer to the average MRI based values *i.e.,* 0.416 m (right) and 0.412 m (left) than *Addbio model i.e.,* 0.422 m. In the case of pelvis, the average estimated length by *Addbio model* for all participants is 0.173 m, which is closer to the MRI-based value of 0.172 m compared to both OpenSim models *i.e.,* symmetric: 0.164 m, asymmetric: 0.167 m. An additional box plot illustrating these results is provided in the Appendix A.

We compared the average RMSE of all markers across all walking trials for the differently scaled models in Table 1. For *OS-Unlock*, the overall average RMSE between virtual and measured markers is lower for the *Addbio model* than the *OS_sym model*, *OS_asym model* and OS model *. For *OS-Lock*, the *OS_sym model* perform better than the *Addbio model*.

**Table 1:** RMSE (cm) between the virtual and experimental markers averaged over three walking trials for all markers.

	OS_asym model	OS_sym model	MRI-based model	AddBio model	OS model *
OS-Unlock	1.685	1.657	1.471	1.585	2.545
OS-Lock	1.592	1.588	1.439	1.725	

The OS model *, designed to exclude the marker registration step, scaled quickly, unlike the *OS_sym model* or *OS_asym model*. However, the RMSE between virtual and experimental markers was highest, when looking at all markers (Table 1) as well as when looking at the leg, foot and torso regions (Tables 2 & 3 & 4). AddBiomechanics showed robustness to changes in the speed compared to the OpenSim. For *OS-Unlock*, we observed 0.01 cm change in AddBiomechanics RMSE *i.e.,* 1.58 cm at 0.8 m/s (slow walking) → 1.59 cm at 1.6 m/s (fast walking), compared to 0.06 cm change in OpenSim RMSE *i.e.,* 1.63 cm (slow walking) → 1.64 cm (fast walking). For *OS-Lock*, the difference in AddBiomechanics RMSE was 0.1 cm *i.e.,* 1.7 cm (slow walking) → 1.8 cm (fast walking) compared to 0.2 cm of OpenSim *i.e.,* 1.5 cm (slow walking) → 1.7 cm (fast walking).

**Table 2:** RMSE (cm) between the virtual and experimental markers averaged over three walking trials for markers attached to the legs.

	OS_asym model	OS_sym model	MRI-based model	AddBio model	OS model *
OS-Unlock	1.531	1.589	1.229	1.442	2.132
OS-Lock	1.339	1.332	1.205	1.592	

**Table 3:** RMSE (cm) between the virtual and experimental markers averaged over three walking trials for markers attached to the feet.

	OS_asym model	OS_sym model	MRI-based model	AddBio model	OS model *
OS-Unlock	1.495	1.323	1.246	1.418	1.591
OS-Lock	1.367	1.372	1.037	1.317	

**Table 4:** RMSE (cm) between the virtual and experimental markers averaged over three walking trials for the markers attached to the torso.

	OS_asym model	OS_sym model	MRI-based model	AddBio model	OS model *
OS-Unlock	2.014	1.937	1.914	2.017	3.537
OS-Lock	2.069	2.066	1.927	2.454	

In Table 2, we examined the marker error of the markers attached to the leg (Appendix A). It was observed that for the *OS-Unlock*, the *Addbio model* are more similar to the *MRI-based model* having a mean difference of 0.213 cm than the *OS_asym model* that showed mean difference of 0.302 cm with the *MRI-based model*. Furthermore, for the *OS-Lock*, *MRI-based model* had greater mean difference of 0.387 cm to the *Addbio model*, in comparison to *OS_sym model* that had mean difference of 0.127 cm.

We further evaluated the error due to the feet and torso markers in Tables 3 & 4. We found that, overall, the *OS_sym model* effectively decreases the torso and feet marker errors for both locked and unlocked groups, with a mean difference of 0.08 cm to *MRI-based model*, in comparison to the *Addbio model* that has a mean difference of 0.267 cm to the *MRI-based model*, except for the feet-markers in *OS-Lock*, the *Addbio model* performs better.

**Figure 4:**
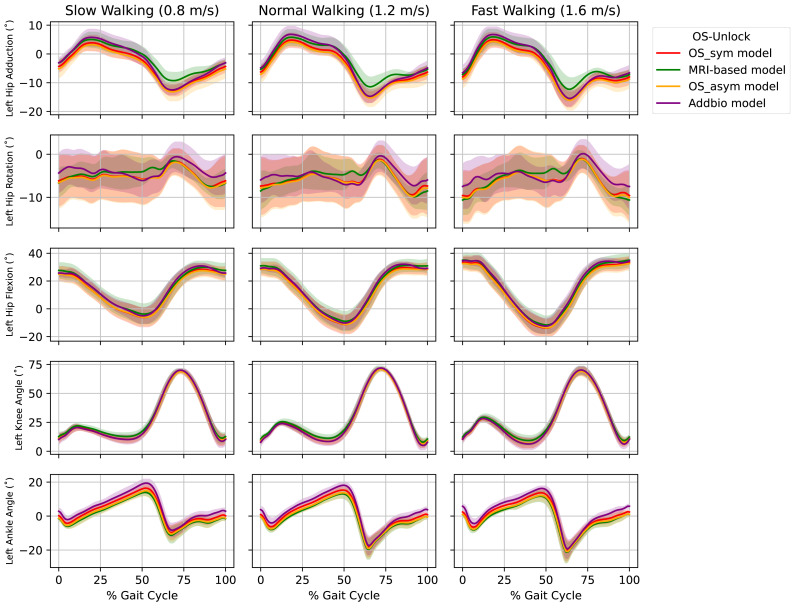
Left leg: Joint angles for all walking trials *i.e.,* slow walking, normal walking and fast walking of each distinctly scaled model. The solid lines shows the joint angles averaged across all participants in OS-Unlock; the shaded region represents the standard deviation across subjects. The maximum angle difference is 6.1° for ankle angle in slow walking.

We analyzed the ankle angle, knee angle and hip flexion angle for both right and left leg. The results of *OS-Unlock* in Fig. 4, shows that for all models the ankle angle, knee angle and the hip flexion angle trajectories were almost similar. The maximum angle difference for the *OS-Unlock* was 6.1°, reported for the left ankle angle of slow walking. The *Addbio model* had consistently the largest difference to the *MRI-based model* while the *OS_asym model* had the lowest difference for the hip flexion, knee and ankle angles. The *MRI-based model* showed difference to other models at 50–80% of gait cycle for the hip adduction and 40–60% of gait cycle for the hip rotation. The maximum angle difference for the *OS-Lock* was 9.2°, reported for the left ankle angle of normal walking in the Appendix B.

## Discussion

We validated musculoskeletal models that were scaled using OpenSim and AddBiomechanics against models personalized using MRI. The pelvis and femur lengths of AddBiomechanics (*OS-Unlock*) closely matched the MRI-lengths (Fig. 3). Furthermore, the RMSE of the leg markers (Appendix A) showed that AddBiomechanics model led to smaller errors than the OpenSim model for the *OS-Unlock* (Table 2). On the other hand, OpenSim performed better in reducing the overall error due to the torso and feet markers for the *OS-Lock* and *OS-Unlock* (Tables 3 & 4). This could be because of the manual registration in OpenSim. AddBiomechanics was more robust in adapting to speed changes compared to OpenSim. Additionally the joint angles of the lower limbs in the Appendix B revealed that the angle trajectories of the AddBiomechanics, OpenSim, and MRI-based models are similar for the knee angle and hip flexion angle in the cases of both the stance and flight phases of walking. There is a slight variation between the different models for the ankle angle, with a maximum angle difference of 9.2°.

This study focused on validating the time-efficient and user independent models scaled by AddBiomechanics to the time-consuming, static pose and operator dependent models scaled by OpenSim. AddBiomechanics provides an automatic scaling of the generic musculoskeletal models in remarkably less time. It took us about an hour, depending on the server load and the internet speed, to process the data of one participant with AddBiomechanics. In comparison, it took us several hours for both the symmetric and asymmetric scaling in OpenSim.

The overall average RMSE, joint angles and the estimated scaled lengths, in light of the MRI data, suggest that AddBiomechanics provides a better alternative for model scaling compared to conventional OpenSim scaling, for tasks involving time constraints. AddBiomechanics automatically performs the scaling and inverse kinematics in a one-step process which makes it independent of the user expertise and user input. However, the marker RMSE was lower for the OpenSim models with locked coordinates than the AddBiomechanics model. For such data, the scaled model can be easily visualized by associating to it the static pose, and any discrepancy can be detected earlier and corrected straight away before initiating a separate inverse kinematics operation in OpenSim. But these models showed highest difference in joint angles to the MRI-based models. Moreover, the OpenSim models generally tends to perform better in reducing the errors contributed to the torso and feet markers.

The marker registration step during scaling in OpenSim, makes the model prone to operator’s subjectivity in adjusting the model markers to better match the experimental markers and choosing different marker weights. This makes it a time consuming process since the operator has to go through a iterative guess-and-check process, based on visual feedback in adjusting the model marker positions, before achieving a desired accuracy. But excluding it will undermine the validity of scaling, as reflected by the results, and therefore a user has to follow the marker registration step in OpenSim. However, AddBiomechanics is free from this user-dependent step, making it more time efficient.

We found that the symmetric scaled models performed almost similar to the asymmetric scaled models. The symmetric scaling in OpenSim uses same scale factors for both right and left sides of the body while the asymmetric scaling uses different scale factors for each side. The asymmetric scaling is better for cases involving different leg lengths data or patient data. As the data in this study was from healthy subjects, it could be the possible reason for the performance-wise close resemblance between both models.

Our results supports the findings of Werling et al. (2023). In their study, the AddBiomechanics based scaling resulted in the reduction of the RMSE for the running dataset to 1.5 cm from the 4.3 cm originally published results Hamner & Delp (2013), that is similar to our average RMSE of the AddBiomechanics for our walking data in Table 1. Their joint angles also look qualitatively similar to our joint angles in the Appendix B.

In a recent study, AST toolkit has been suggested to reduce the time taken by scaling a generic musculoskeletal model in OpenSim (Di Pietro et al., 2024). We did not compare our results with this toolkit because it is dependent on the manual intervention of the operator in creating the scaling setup file with as much accuracy as possible. It is not a standalone software and relies on valid MATLAB license and correct configuration of the OpenSim API *via* MATLAB. Furthermore, AST was tested only on a sample of male data. In comparison, AddBiomechanics is an open-source web based software, which makes it easier for everyone to work, without requiring technical expertise. However, AddBiomechanics depends on public data sharing and publication on a US institution server. And this can pose challenges in certain research and clinical settings.

The application of neural networks for the prediction of various biomechanical parameters suffers from dataset size limitation. AddBiomechanics has the potential to act as a standardization scaling tool through which potential errors due to scaling in various datasets could be minimized. These uniformly scaled datasets could be later combined into a larger dataset *i.e., AddBiomechanics Dataset 1.0*: comprising over 70 h of data, from 15 sources, with 12 h clinical-grade data (Werling et al., 2024). In future, such unified resources could potentially pave the way for robust utilization of deep learning in biomechanics.

The goal of the study was to explore MRI-based evaluation and validation of the length-based scaling approach for healthy individuals, including both males and females. MRI-based models have certain limitations. These models are computationally costly to develop and manually defining the anatomical landmarks by an operator could introduce variability. For consistency during this study, the same operator defined these landmarks across all participants. AddBiomechanics scales segment lengths, but does not account for variations in bone shape, which can impact the precision of simulations results. Moreover, the preliminary observations of the study presented are only for the inverse kinematics. It will be interesting to examine if the scaling of the segment’s mass shows the same trend as that of the segment’s length and the impact of scaling pelvis in multiple dimensions, given its multiple muscle attachment sites. The study can be expanded with validation from EMG data to perform inverse dynamics and muscle force estimations as well. In future, the framework still needs to be evaluated for clinical applications.

## Conclusions

From our exploratory study into MRI-based validation of MSK models, we conclude that AddBiomechanics can serve as a viable alternative to OpenSim for length-based scaling a musculoskeletal model. Our results indicate comparable accuracy with no meaningful differences. Considering the time and effort involved, AddBiomechanics appears to be more practical, especially since our study results also support its use.

## Supplemental Information

10.7717/peerj.21114/supp-1 Supplemental Information 1 Marker list, marker pairs, and segment length box plot

10.7717/peerj.21114/supp-2 Supplemental Information 2 Right and Left Leg Joint Angle Profiles

## Additional Information and Declarations

### Data Availability

The following information was supplied regarding data availability: The raw data is available at Zenodo: Rauf, N., Amon, P., Stuprich, C., Laun, F. B., & Koelewijn, A. (2025). Magnetic Resonance Imaging Validation of Length-Based Scaling of Musculoskeletal Models Using Opensim and Addbiomechanics for Walking [Data set]. Zenodo. https://doi.org/10.5281/zenodo.17591604
