# Supplementary material for: Exploring magnetic resonance imaging validation of length-based scaling of musculoskeletal models using OpenSim and AddBiomechanics for walking
Source: PeerJ. 2026 Apr 22;14:e21114. doi: 10.7717/peerj.21114 (PMC13109979; doi:10.7717/peerj.21114)
Supplement: Supplemental Information 1 [file peerj-14-21114-s001.pdf]

A COMPLETE LIST OF ALL 41-MARKERS, WITH THE 16 LEG-MARKERS (HIGHLIGHTED IN BOLD).

- Chest
- SpineThoracic12
- LShoulderTop
- RShoulderTop
- **RAsis**
- **LAsis**
- **RFemurHead**
- **LFemurHead**
- **RThighFrontHigh**
- **LThighFrontLow**
- **RKneeOut**
- **RKneeIn**
- **LKneeOut**
- **LKneeIn**
- **RShinFrontHigh**
- **LShinFrontLow**
- **RAnkleOut**
- **RAnkleIn**
- **LAnkleOut**
- **LAnkleIn**
- LToe
- LHeelBack
- LForefoot1
- RToe
- RHeelBack
- RForefoot1
- LPsis
- RPsis
- LUArmLow
- LElbowIn
- LElbowOut
- LWristIn

- LWristOut
- RUArmHigh
- RElbowOut
- RElbowIn
- RWristIn
- RWristOut
- STRN
- NAVE
- SACR

Table A1. Different marker pairs used for scaling a body segment

Segment	Marker pair			
Torso	SACR	STRN	SACR	R/LShoulderTop
Pelvis	RAsis	LAsis		
Thigh	RAsis	RKneeOut	LAsis	LKneeOut
Shank	RkneeOut	RAnkleOut	LKneeOut	LAnkleOut
foot	RHeelBack	RToe	LHeelBack	LToe

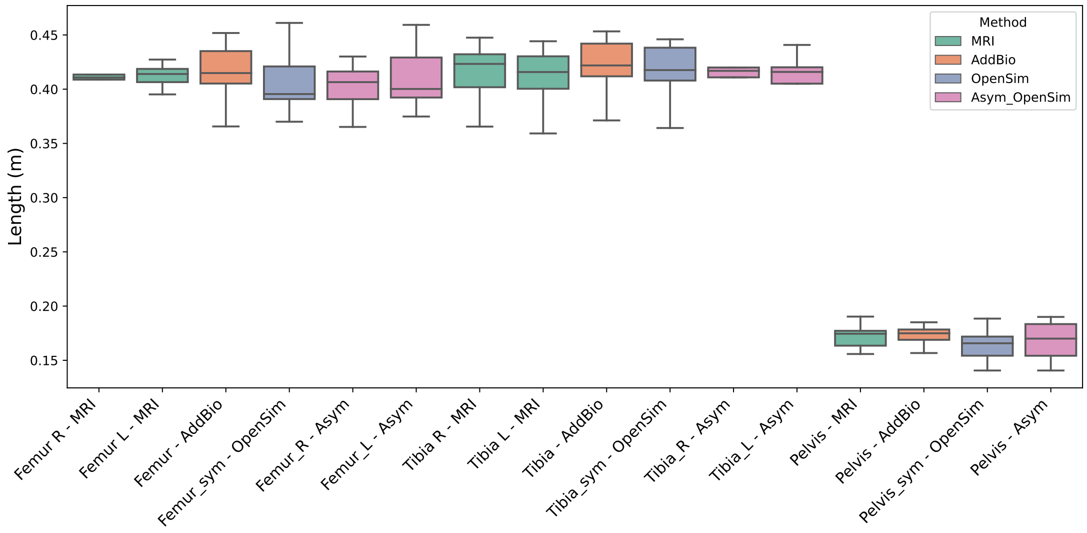

Figure A1. An additional Box-plot for the segment lengths estimated by AddBiomechanics and OpenSim models.
