# Supplementary material for: Exploring magnetic resonance imaging validation of length-based scaling of musculoskeletal models using OpenSim and AddBiomechanics for walking
Source: PeerJ. 2026 Apr 22;14:e21114. doi: 10.7717/peerj.21114 (PMC13109979; doi:10.7717/peerj.21114)
Supplement: Supplemental Information 2 [file peerj-14-21114-s002.pdf]

B ANKLE ANGLE, KNEE ANGLE AND HIP FLEXION ANGLE PLOTS FOR THE RIGHT AND LEFT LEG.

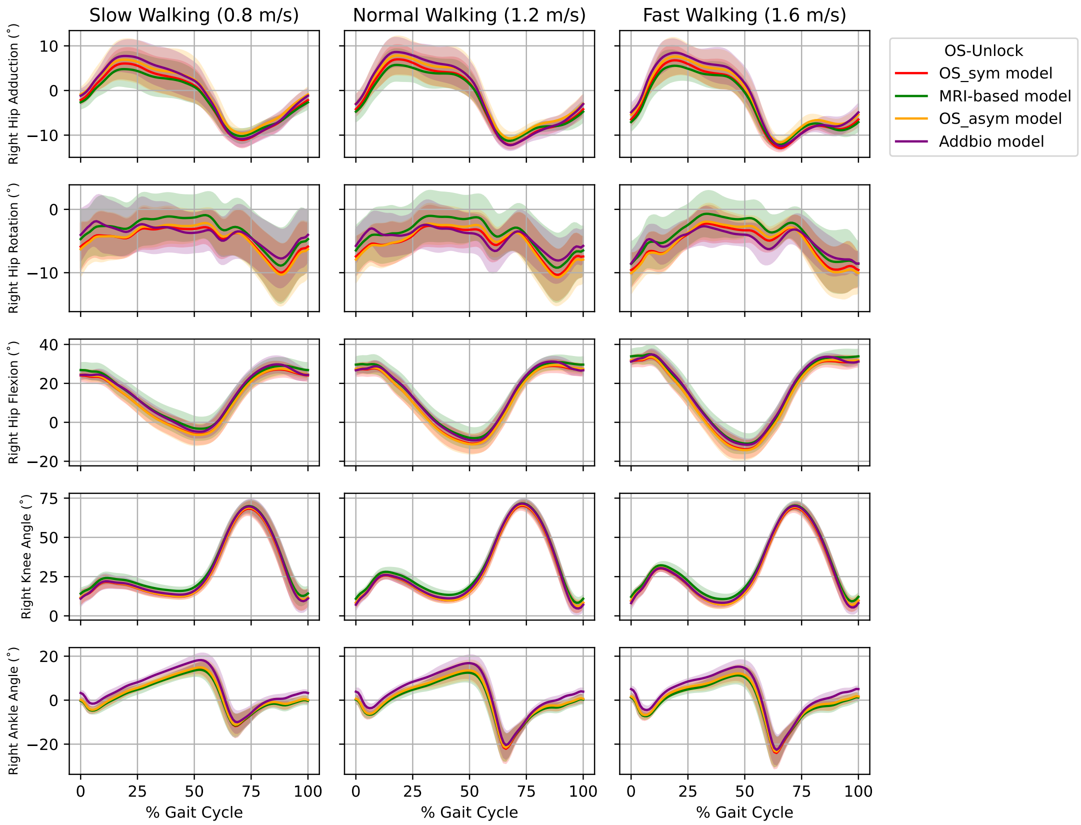

Figure B1. Right leg: Joint angles for all walking trials, i.e., slow walking, normal walking and fast walking of each distinctly scaled model. The solid lines shows the joint angles averaged across all participants in OS-Unlock; the shaded region represents the standard deviation across subjects. The maximum angle difference is 4.73°for ankle angle in slow walking.

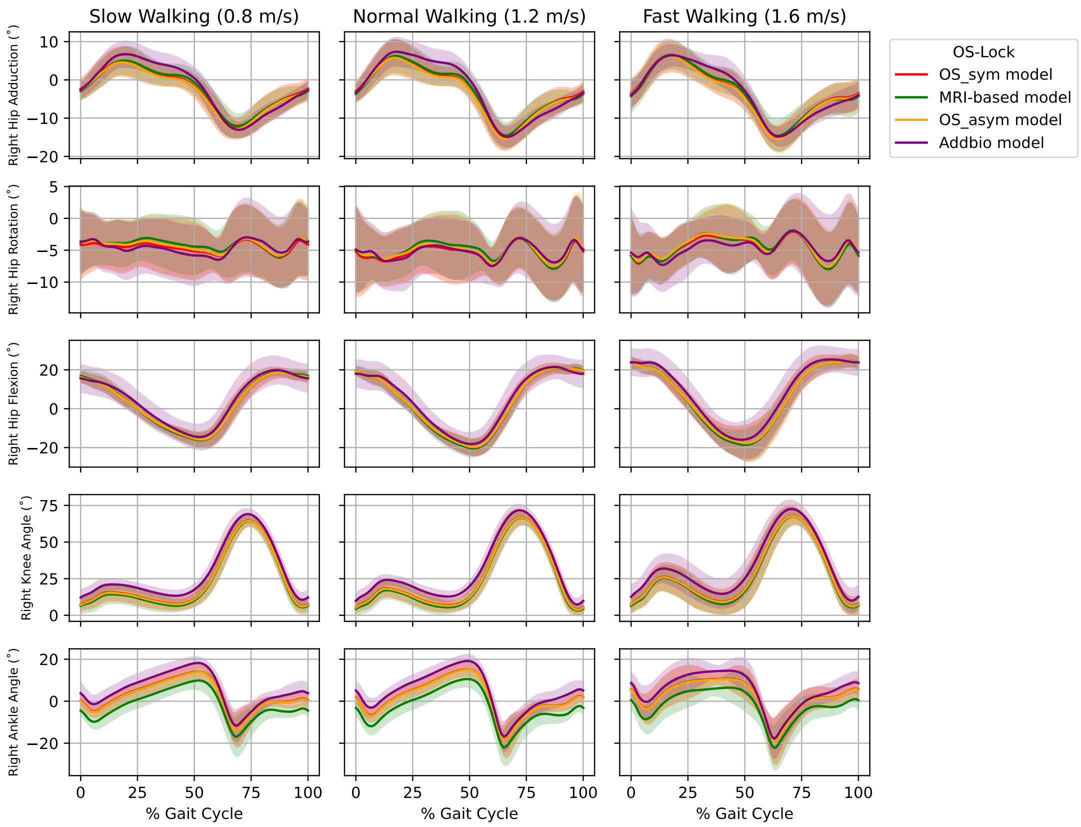

Figure B2. Right leg: Joint angles for all walking trials, i.e., slow walking, normal walking and fast walking of each distinctly scaled model. The solid lines shows the joint angles averaged across all participants in OS-Lock; the shaded region represents the standard deviation across subjects. The maximum angle difference is 9.2°for ankle angle in slow walking.

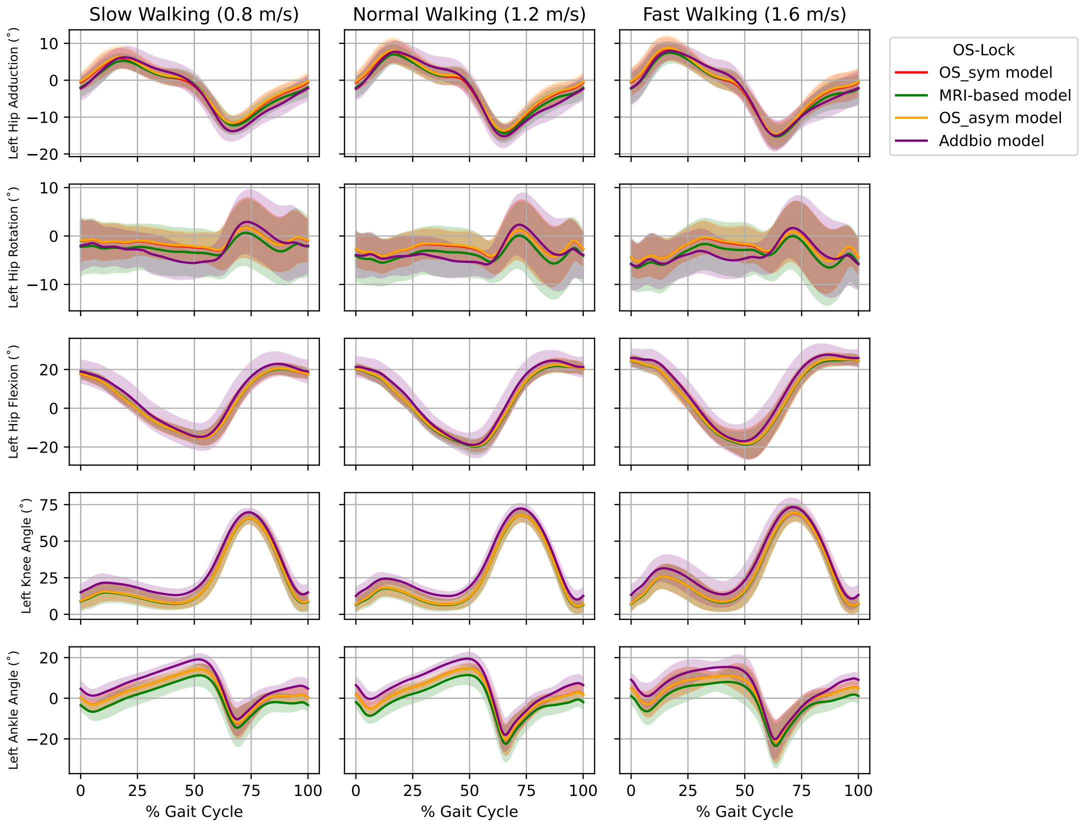

Figure B3. Left leg: Joint angles for all walking trials, i.e., slow walking, normal walking and fast walking of each distinctly scaled model. The solid lines shows the joint angles averaged across all participants in OS-Lock; the shaded region represents the standard deviation across subjects. The maximum angle difference is 8.4°for ankle angle in slow walking.
